# LAPAROSCOPIC INGUINAL HERNIA REPAIR: THE LONG-TERM ASSESSMENT OF CHRONIC PAIN AND QUALITY OF LIFE

**DOI:** 10.1590/0102-672020220002e1695

**Published:** 2022-11-14

**Authors:** Gustavo Rodrigues Alves Castro, Andressa Zilles, Larissa Dill Gazzola, Renar Brito Barros, José Alfredo Sadowski, Camila Roginski Guetter

**Affiliations:** 1 Faculdades Pequeno Príncipe – Curitiba (PR), Brazil; 2 Sugisawa Hospital – Curitiba (PR), Brazil; 3 Johns Hopkins Bloomberg School of Public Health – Baltimore (MD), United States of America.

**Keywords:** Hernia, Hernia, Inguinal, Herniorrhaphy, Chronic Pain, Quality of Life, Hérnia, Hérnia Inguinal, Herniorrafia, Dor Crônica, Qualidade de Vida

## Abstract

**BACKGROUND:**

Laparoscopic approaches to inguinal hernia repair include transabdominal extraperitoneal and transabdominal preperitoneal, both of which are widely performed and employ mesh. Indicators of success for these surgical procedures include incidence of complications, time to return to daily activities, incidence of postoperative chronic pain, and the long-term postoperative patient satisfaction.

**OBJECTIVE:**

This study aimed to evaluate and compare long-term postoperative incidence of chronic pain and overall quality of life among patients undergoing transabdominal extraperitoneal or transabdominal preperitoneal inguinal hernia repair.

**METHODS:**

This was a retrospective cross-sectional study. Medical records were analyzed, and the SF-36 questionnaire and Visual Analog Scale were applied to assess quality of life and chronic pain in patients undergoing laparoscopic inguinal hernia repair between January 2017 and February 2021.

**RESULTS:**

A total of 167 patients status post laparoscopic inguinal hernia repair, who were 3 months postoperatively or longer, were included in the study. Among the early complications seen, seroma was most common in the transabdominal preperitoneal group (p=0.04). Subsequently, 40 of the initial 167 patients answered to the survey instrument (SF-36 and Visual Analog Scale). Mean patient-reported pain (Visual Analog Scale score) was statistically similar between groups, with 1.29 for transabdominal preperitoneal and 1.68 for transabdominal extraperitoneal (p=0.92). In the domains evaluated by the SF-36, there was no significant difference between the samples.

**CONCLUSION:**

Both transabdominal extraperitoneal and transabdominal preperitoneal techniques for hernia repair have similar results in the late postoperative period regarding quality of life and prevalence of chronic pain. They are also comparable in terms of major early postoperative complications, except for seroma, with a higher incidence in patients undergoing transabdominal preperitoneal.

## RESUMO

**RACIONAL:**

Atualmente o tratamento da hérnia inguinal pode ser laparoscópico totalmente extraperitoneal ou transabdominal pré-peritoneal, ambas as técnicas utilizam tela e são amplamente empregadas. O sucesso do procedimento cirúrgico é medido pelo menor número de complicações, retorno rápido às atividades cotidianas, presença ou não de dor crônica e pelo grau de satisfação do paciente no pós-operatório.

**OBJETIVOS:**

Avaliar e comparar a presença de dor crônica e qualidade de vida no pós operatório tardio de pacientes submetidos às duas técnicas de cirurgias laparoscópicas de correção de hérnia inguinal.

**MÉTODOS:**

O estudo consistiu em uma abordagem quantitativa transversal retrospectiva. Foi realizada análise de prontuários e aplicação de questionário SF-36 e Escala Visual Analógica para avaliação de qualidade de vida e dor crônica em pacientes submetidos a hernioplastia inguinal laparoscópica entre janeiro de 2017 a fevereiro de 2021.

**RESULTADOS:**

Foram analisados 167 prontuários com mais de 3 meses de pós-operatório. Dentre as complicações precoces avaliadas, o seroma foi mais comumente diagnosticado no grupo transabdominal pré-peritoneal (p=0,04). Posteriormente, 40 dos 167 pacientes responderam ao instrumento de pesquisa composto por SF-36 e Escala Visual Analógica. A média para dor crônica, mensurada através da Escala Visual Analógica, foi de 1,29 para transabdominal pré-peritoneal *versus* totalmente extraperitoneal com 1,68 (p=0,92). Já nos domínios avaliados pelo SF-36 não houve diferença significativa entre as amostras (p>0,05).

**CONCLUSÕES:**

As técnicas totalmente extraperitoneal e transabdominal pré-peritoneal apresentam resultados semelhantes no pós-operatório tardio com base na qualidade de vida e dor crônica. As técnicas também são comparáveis em termos das principais complicações pós-operatórias precoces, exceto para seroma, que apresentou maior incidência em pacientes submetidos a transabdominal pré-peritoneal.

## INTRODUCTION

Hernia can be defined as an abnormal protrusion of an organ or tissue resulting from a deformity in the wall of the cavity containing it. Inguinal hernias can be repaired through open procedures or laparoscopic procedures. Currently, the laparoscopic approach can be transabdominal extraperitoneal (TEP) or transabdominal preperitoneal (TAPP), both of which use mesh and are widely performed^3,10,11^.

Both are minimally invasive and can be performed easily and with smaller incisions. In addition, they allow improvements in quality of life, combined with a faster recovery period, reduction of postoperative pain, and morbidity^1,17^.

Pain is common postoperatively; however, it is expected to occur in the first few days and to respond well to analgesics with progressive improvement, ceasing completely in a few weeks. Nevertheless, persistence of pain for a period longer than 3 months can be characterized as chronic pain. Postoperative pain is an important index, as it has direct implications on morbidity, healthcare-associated costs, and quality of life. It is, therefore, relevant to be evaluated in the surgical practice^6,9^.

Given the improvement of minimally invasive surgery techniques with lower complication rates, surgical outcomes are increasingly dictated by patient-reported postoperative satisfaction and their perception of how they feel when performing daily activities. This type of postoperative quality-of-life assessment using a variety of tools has become the main way of measuring parameters of therapeutic success^4^.

The aim of this study was to compare laparoscopic techniques of inguinal hernia repair, considering the two most used methods (i.e., TEP and TAPP), with respect to quality of life and prevalence of chronic pain in the late postoperative period. Secondary objectives include evaluation of epidemiological data on patients with inguinal hernias and occurrence of early postoperative complications.

## METHODS

This is a retrospective cross-sectional study, approved by the Institutional Review Board of the Pequeno Príncipe Faculty (CEP/FPP, number 4.259.587). This study consisted in the evaluation of medical records of 167 patients who underwent unilateral or bilateral laparoscopic inguinal hernia repair between January 2017 and February 2021. Subsequently, patients included in the study were contacted to respond to the survey tool for quality of life (SF-36) and for chronic pain (Visual Analog Scale — VAS). Consent was obtained.

All study participants were at least 3 months postoperatively at the time of data collection. Study participants were operated on by the same team of general surgeons and gastrointestinal surgeons. All surgical procedures were performed under general anesthesia. In patients undergoing TAPP, pneumoperitoneum was performed, followed by placement of three trocars for optic access and two for laparoscopic forceps. The preperitoneal space was accessed by opening the parietal peritoneum of the affected inguinal region, followed by dissection and identification of the local anatomy. The hernia was localized, its contents and sac were reduced. Afterward, a 15´15 cm polypropylene mesh was positioned covering the entire region of the myopectineal space and secured with 4–5 endostapler staples. The parietal peritoneum was closed with continuous suture with long-lasting absorbable suture and the abdomen was deflated. Skin was closed with nonabsorbable suture.

For patients undergoing TEP, the suprapubic preperitoneal space was punctured with a Veress needle and then three trocars were positioned, one for optics and two for laparoscopic forceps. The preperitoneal space was dissected and the local anatomy was identified. The hernia was localized, its contents and sac were reduced, and a 15´15 cm polypropylene mesh was positioned covering the entire region of the myopectineal space and secured with 4–5 endostapler staples. The preperitoneal space was deflated and the skin was closed with nonabsorbable suture.

The clinical variables analyzed in patient’s medical records were age, sex, time from surgery, comorbidities, surgical technique for repair (TEP or TAPP), and recent postoperative complications. The postoperative complications evaluated for this study were hematoma, seroma, surgical site infection, and ischemic orchitis.

The Charlson Comorbidity Index (CCI) was used to assess comorbidities, analyzing 19 clinical conditions, including cardiovascular disease, diabetes mellitus, liver disease, and lung disease. This index aims to predict risk of death related to having comorbidities^13,18^.

Patients excluded from this study included patients under 18 years of age, time from surgery less than 3 months, open approach to hernia repair, repair of recurrent hernias or femoral hernias, and those for whom the repair was indicated in urgent or emergency situations (incarcerated or strangulated inguinal hernias).

The SF-36 questionnaire was used in eight domains: functional capacity, physical aspects, pain, general health status, vitality, social aspects, emotional aspects, and mental health^6,7,12^.

The tool utilized to assess postoperative pain in this study was the VAS. The VAS is a visual, simple, and effective validated tool for pain assessment. It consists of a line with ends numbered from 0 to 10, in which the patient assigns a score to their pain, with 0 being no pain and 10 being the worst pain imaginable^16^.

Finally, patients who filled out the questionnaire incorrectly, those who could not be contacted (via phone or email), or those who declined to participate in the study were excluded from the analysis.

### Statistical Analysis

Data collected from the two groups (TAP and TEP) were analyzed using mean and standard deviation for continuous variables and absolute and relative frequencies for categorical and ordinal variables. Shapiro-Wilk test and histogram generation were used for sample distribution analysis. Student’s t-test for normal continuous dependent variables, Mann-Whitney test for non-normal continuous dependent variables, and chi-square test for categorical dependent variables were also used. A significance level of 5% was used for this study.

## RESULTS

A total of 167 patients who underwent laparoscopic inguinal hernia repair were included in the study. Data on patient demographics and early postoperative complications were analyzed. Subsequently, the patients were contacted and only 40 responded to the survey tool composed of SF-36 and VAS (18 patients in the TAPP group and 22 in the TEP group).

### Epidemiological Profile

Among the 167 medical records that were analyzed, only 12 (7.19%) patients were female. The mean age of the study participants was 55.19 years, with the oldest patient being 86 years old and the youngest being 22 years old. There was no statistically significant difference between the TAPP and TEP groups in terms of age (p=0.33). In all 46 (27.54%) patients had medical history notable for hypertension, 24 (14.37%) for dyslipidemia, 19 (11.38%) for diabetes, 9 (5.39%) for benign prostatic hyperplasia, 3 (1.80%) had a previous myocardial infarction, and 4 (2.40%) had a previous stroke.

The groups were statistically balanced regarding comorbidities according to the CCI (p=0.61). Mean CCI was 1.21±0.16 for the TAPP group and 1.33±0.15 for the TEP group.

### Complications

Among the early postoperative complications, seroma was the most prevalent, which was present in 16 (9.58%) patients. This was followed by hematoma in 8 (4.79%) cases, and ischemic orchitis and pain, both of which occurred in 3 (1.80%) patients. After statistical analysis, seroma was found to be significantly more commonly diagnosed in TAPP patients as compared to TEP (p=0.04), while the other complications showed no significant difference between groups (p>0.05) (Table 1).

**Table 1:** Demographic data (transabdominal extraperitoneal vs. transabdominal preperitoneal).

		TEP (n=92)	TAPP (n=75)	p-value
Sex	Female	5	7	0.33
	Male	87	68	
Age		56.38±1.44	54.12±1.77	0.2
Complications	Hematoma	3 (3.3%)	5 (6.7%)	0.47
	Seroma	5 (5.4%)	11 (14.7%)	0.04
	Ischemic orchitis	2 (2.2%)	1 (1.3%)	>0.05
	Postoperative pain	1 (1.19%)	2 (2.7%)	0.59
CCI		1.33±0.5	1.21±0.16	0.60

TEP: transabdominal extraperitoneal; TAPP: transabdominal preperitoneal; CCI: Charlson Comorbidity Index.

### Quality of life

**Figure 1:**
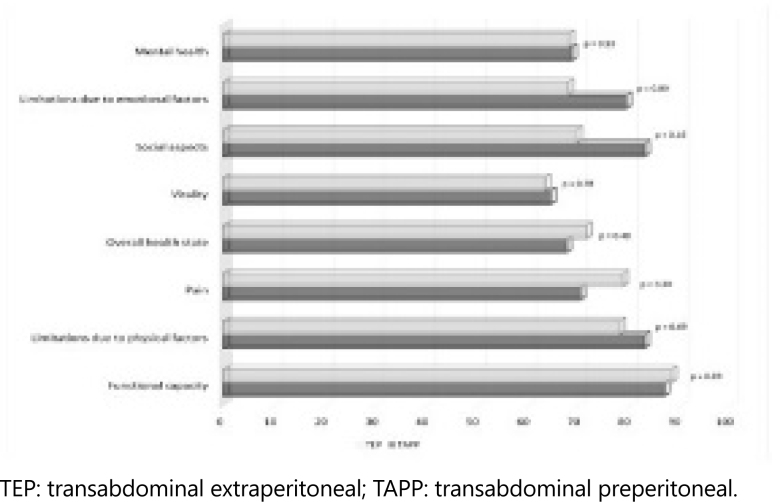
**Domains SF**-36, transabdominal extraperitoneal vs. transabdominal preperitoneal.

Analysis of the results of the SF-36 survey, responded by 40 patients, revealed no significant difference between the evaluated domains: mental health, limitations due to emotional and social aspects, vitality, overall health state, pain, limitation due to physical factors, and functional capacity (Figure 1).

The lowest averages were seen in the vitality domain, in which questions were based on whether patients felt vigorous, with will, strength, energy, and whether there was presence of exhaustion and/or fatigue. Meanwhile, the highest averages were seen in the functional capacity domain, in which the questions were related to basic daily activities, such as lifting heavy objects, carrying groceries, climbing stairs, kneeling, walking a few blocks, showering, and getting dressed.

#### Chronic Pain

**Figure 2:**
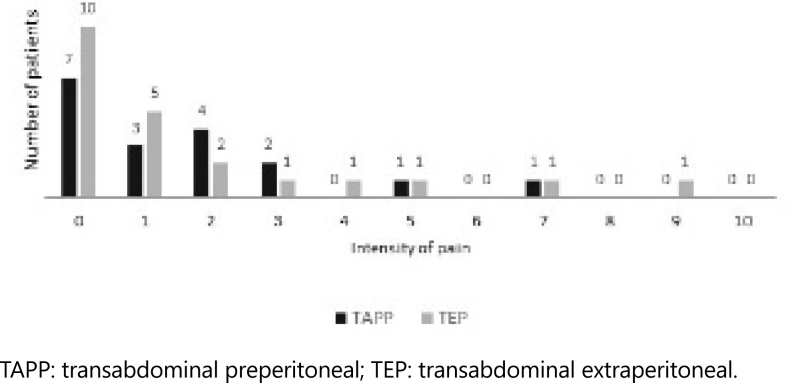
Intensity of pain by the Visual Analog Scale.

When analyzing the results of the VAS, 17 (42.50%) patients answered the survey and reported zero pain intensity, 23 patients reported some pain, but only 3 patients had pain intensity greater than 5 (Figure 2). No statistically significant difference was observed between TEP and TAPP (p=0.71). The mean pain score identified by patients was 1.61 for TAPP and 1.68 for TEP.

## DISCUSSION

Regarding postoperative complications, the incidence of seroma was significantly higher in the TAPP group (Table 1). These results are similar to those found by Köckerling et al.^14^, who carried out with 2246 patients (p<0.001).

There was no significant difference between TEP and TAPP techniques with regard to the incidence of postoperative hematoma, postoperative pain, and ischemic orchitis (Table 1). This is in agreement with the study by Aiolfi et al.^2^. In their systematic review and meta-analysis, 9 studies with a total of 54,138 patients were evaluated, and results suggested that the open, TAPP, and TEP techniques are comparable in terms of postoperative hematoma, seroma, recurrence, infectious complications, urinary retention, and length of hospital stay.

The SF-36 questionnaire consists of a 36-item generic health survey designed to measure functional health and well-being on a scale (percentage) from 0 (suboptimal) to 100 (optimal)^19^. Results for the TAPP and TEP groups were similar in all domains of the scale, showing no significant difference between the techniques (Figure 1).

The prospective randomized controlled study carried out by Bansal et al.^5^ with 314 patients showed that both TAPP and TEP techniques improved patients’ quality of life, from comparing preoperative to 3 months postoperatively. The TEP group showed significant improvement in all domains (i.e., mental health, vitality, emotional pain, overall health, pain, emotional, social, and physical functions). The TAPP group showed significant improvement in all domains, except those of vitality and social functions. However, both groups were comparable postoperatively in terms of quality of life.

The Brazilian cohort study of 67 patients by Maliska et al.^15^ compared quality of life using the SF-36 survey between preoperative and postoperative periods (3 month postoperative), after open hernia repair. A significant improvement was observed in the domains of physical aspect and pain, and significant differences were seen in the domains of functional capacity, mental health, social aspects, and emotional aspects. Only the domains of overall health and vitality did not demonstrate a statistically significant improvement (p>0.05). A similar result was found in our study in the late postoperative period of TEP and TAPP, in which the lowest means were obtained in the domains of vitality, mental health, and overall health state, while the highest mean was also in the domain of functional capacity (Figure 1). Our results are similar to the studies mentioned. However, there is a lack of studies that utilize the SF-36 scale as a parameter for evaluating quality of life comparing TAPP and TEP inguinal hernia repair techniques.

Regarding chronic pain, present 3 months after surgery, and assessed using the VAS, there was no difference between TEP and TAPP (Figure 2). The results of this study were similar to those of Bansal et al.^4^, who observed that the TAPP group required more analgesia in the early postoperative period and also had a higher pain score in the first 6 and 24 h after surgery. However, regarding moderate-to-severe chronic pain, there was no significant difference between the two techniques: 1.25% of the TEP group and 1.29% of the TAPP group had a score of >3 on the VAS.

Donovan et al.^8^ retrospectively analyzed 77 patients, with 40 undergoing TAPP approach and 37 undergoing TEP approach, and found that there was a significant difference in the use of opioids for pain relief in the immediate postoperative period. In the TEP technique, the time of use of opioids (in days) was shorter than in TAPP (0 vs. 3; p=0.007). However, there was no significant difference in the presence of chronic pain between the TAPP and TEP techniques (p=0.772).

It is worth mentioning that due to the SARS-CoV-2 pandemic, social distancing, and scarcity of human resources and medical supplies, the number of elective surgeries performed in 2020 was significantly lower than in previous years. In addition, getting in contact with patients to carry out this study was done exclusively via telephone, so the insecurity of sharing information and experiences at a distance were factors that could have limited the number of patients participating in the study. The complexity of the SF-36 questionnaire could have also led to a smaller number of participants. In addition, limitations of this study are also related to data being acquired retrospectively and from patients operated in a single center.

Despite these limitations, this study stands out for being one of the first to present national data comparing quality of life and chronic postoperative pain between the two main laparoscopic surgical techniques for inguinal hernia repair.

## CONCLUSION

Patients undergoing TAPP inguinal hernia repair in this study had higher incidence of postoperative seroma as compared to those undergoing TEP repair. However, the techniques are comparable in terms of incidence of other early postoperative complications. In addition, no significant difference was seen between TEP and TAPP in quality of life and chronic pain in the late postoperative period.

It is important to emphasize that further research is needed to establish whether there are any significant differences between the techniques, given that our sample is limited and consists of patients operated on in a single institution. Therefore, the choice of technique should take into consideration the surgeon’s experience and the patient’s overall clinical status and presentation.

## References

[1] Aghayeva A, Benlice C, Bilgin IA, Bengur FB, Bas M, Kirbiyik E (2020). Laparoscopic totally extraperitoneal vs robotic transabdominal preperitoneal inguinal hernia repair: assessment of short- and long-term outcomes. Int J Med Robot.

[2] Aiolfi A, Cavalli M, Micheletto G, Lombardo F, Bonitta G, Morlacchi A (2019). Primary inguinal hernia: systematic review and Bayesian network meta-analysis comparing open, laparoscopic transabdominal preperitoneal, totally extraperitoneal, and robotic preperitoneal repair. Hernia.

[3] Bakri M, Lovato FC, Diosti GM, Salles YLSG, Moreira PHB, Collaço LM (2022). Comparative analysis of tissular response after abdominal wall repair using polypropylene mesh and bovine pericardium mesh. ABCD Arq Bras Cir Dig.

[4] Bansal VK, Krishna A, Manek P, Kumar S, Prajapati O, Subramaniam R (2017). A prospective randomized comparison of testicular functions, sexual functions and quality of life following laparoscopic totally extra-peritoneal (TEP) and trans-abdominal pre-peritoneal (TAPP) inguinal hernia repairs. Surg Endos.

[5] Bansal VK, Misra MC, Babu D, Victor J, Kumar S, Sagar R (2013). A prospective, randomized comparison of long-term outcomes: chronic groin pain and quality of life following totally extraperitoneal (TEP) and transabdominal preperitoneal (TAPP) laparoscopic inguinal hernia repair. Surg Endosc.

[6] Bittner R, Köckerling F, Fitzgibbons RJ, LeBlanc KA, Mittal KM, Chowbey P (2018). Laparo-endoscopic hernia surgery: evidence based clinical practice.

[7] Ciconelli RM, Ferraz MB, Santos W, Meinão I, Quaresma MR, Rodrigues M (1999). Tradução para a língua portuguesa e validação do questionário genérico de avaliação da qualidade de vida SF-36 (Brasil SF-36). Rev Bras Reumatol.

[8] Donovan K, Denham M, Kuchta K, Carbray J, Ujiki M, Linn J (2020). Laparoscopic totally extraperitoneal and transabdominal preperitoneal approaches are equally effective for spigelian hérnia repair. Surg Endosc.

[9] Fontenele SC, Da Silva ER (2018). Prevalência de dor crônica pós-hernioplastia inguinal em um hospital universitário. Jornal de Ciências da Saúde – JCS HU-UFPI.

[10] Gomes CA, Gomes FC, Podda M, Braga APF, Ribeiro SC, Vaz LF (2022). Liechtenstein versus laparoscopic transabdominal preperitoneal (TAPP) hernia repair: a prospective comparative study focused on postoperative outcomes in a general surgery unit. ABCD Arq Bras Cir Dig.

[11] Goulart A, Martins S (2015). Hérnia inguinal: anatomia, patofisiologia, diagnóstico e tratamento. Revista Portuguesa de Cirurgia.

[12] Hays RD, Sherbourne CD, Mazel RM (1993). The RAND 36-Item Health Survey 1.0. Health Econ.

[13] Huang YQ, Gou R, Diao YS, Yin QH, Fan WX, Liang YP (2014). Charlson comorbidity index helps predict the risk of mortality for patients with type 2 diabetic nephropathy. J Zhejiang Univ Science B.

[14] Köckerling F, Bittner R, Kuthe A, Hukauf M, Mayer F, Fortelny R (2017). TEP or TAPP for recurrent inguinal hernia repair-register-based comparison of the outcome. Surg Endosc.

[15] Maliska G, Mello ALP, Amaral RP, Bischoff C (2019). Avaliação do impacto da dor crônica na qualidade de vida dos pacientes antes e após hernioplastia inguinal. Revista de Medicina.

[16] Martinez JE, Grassi DC, Marques LGM (2011). Análise da aplicabilidade de três instrumentos de avaliação de dor em distintas unidades de atendimento: ambulatório, enfermaria e urgência. Rev Bras Reumatol.

[17] Teixeira FMC, Pires FPAA, Lima JSF, Pereira FLC, Silva CAS, Paula MHS (2017). Estudo de revisão da cirurgia de hernioplastia inguinal: técnica de Lichtenstein versus laparoscópica. Rev Med Minas Gerais.

[18] Tominaga T, Nonaka T, Takeshita H, Kunizaki M, Sumida Y, Hidaka S (2018). The charlson comorbidity index as an independent prognostic factor in older colorectal cancer patients. Indian J Surg.

[19] Wennergren JE, Plymale M, Davenport D, Levy S, Hazey J, Perry KA (2016). Quality-of-life scores in laparoscopic preperitoneal inguinal hernia repair. Surg Endosc.

